# Supplementary material for: CircRNAs and miRNAs: Key Player Duo in Breast Cancer Dynamics and Biomarkers for Breast Cancer Early Detection and Prevention
Source: Int J Mol Sci. 2024 Dec 4;25(23):13056. doi: 10.3390/ijms252313056 (PMC11641287; doi:10.3390/ijms252313056)
Supplement: Supplementary file 1 [file ijms-25-13056-s001.zip › ijms-3306344-supplementary.pdf]

Supplementary files

Figure S1. KM Survival Analysis of miRNAs Selected for Proposed mRNA/circRNA/miRNA Axes

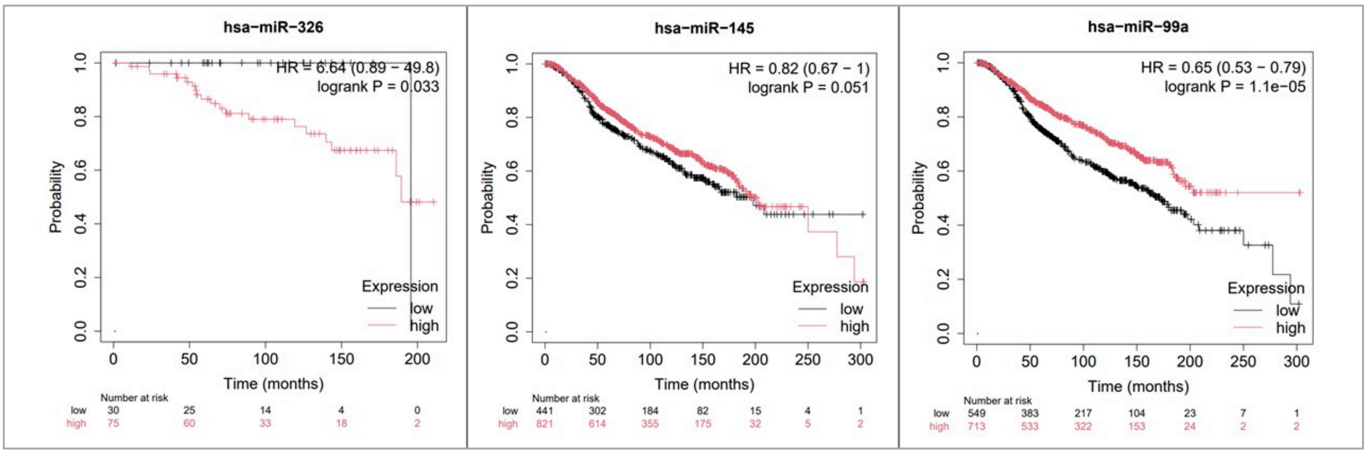

KM survival plots for selected miRNAs associated with the proposed mRNA/circRNA/miRNA axes for early detection and prevention of BC. Each plot correlates the survival probability of BC patients to expression levels (high vs. low) of the specified miRNAs. Survival curves were analyzed using KM Plotter, and significance was determined by log-rank tests, as indicated on each plot.

Figure S2. Enrichment Analysis of miRNAs Selected for Proposed mRNA/circRNA/miRNA Axes

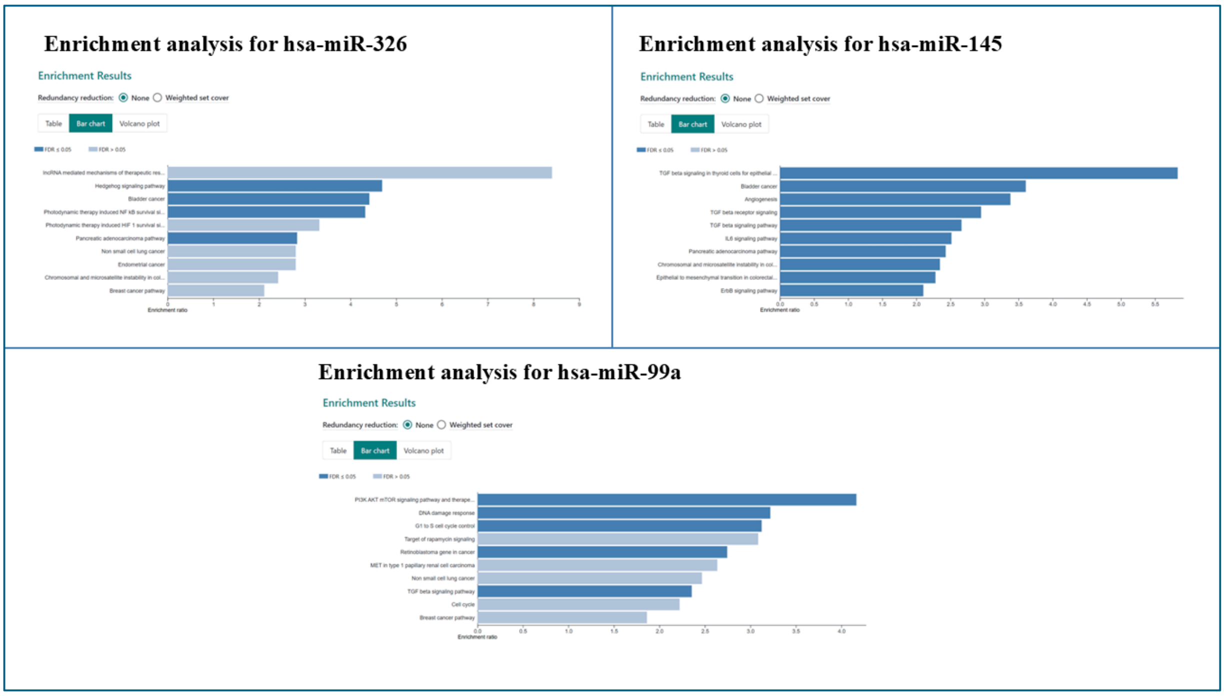

The enrichment analysis results for selected miRNAs within cancer-related pathways, performed using WebGestalt. Each bar represents a pathway significantly enriched with target genes of the specified

miRNAs, identified based on their involvement in BC-related processes. Pathways are ranked by enrichment ratio, indicating the strength of association between each pathway and the miRNA targets. The bars represent the enriched pathways with FDR-corrected p-values, highlighting statistically significant associations.

Figure S3. Differential Expression Analysis of Parental Genes in Breast Cancer using UALCAN.

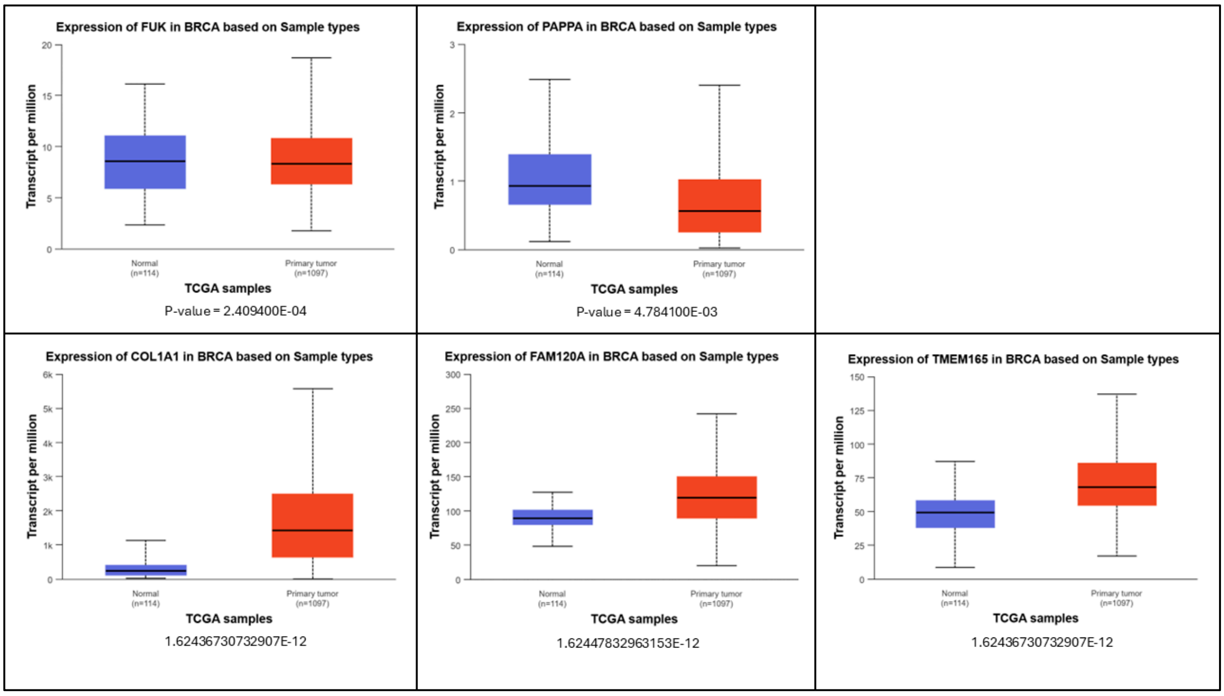

The differential expression of six genes in BC tissues by comparing normal and primary tumor samples are obtained from the TCGA database. Each boxplot displays the expression levels of a specific gene, measured in transcripts per million, with comparisons made between normal samples (n=114) and primary tumor samples (n=1097). Notably, FUK shows significantly lower expression in tumor tissue compared to normal tissue, with a p-value of 2.409400E-04. Similarly, PAPPA is downregulated in tumor samples, with a p-value of 4.784100E-03. COL1A1 exhibits a markedly increased expression in tumor tissue, indicated by a highly significant p-value of 1.62436730732907E-12. The gene FAM120A also shows higher expression in tumor samples, with a p-value of 1.6247832963153E-12. Additionally, TMEM165 is upregulated in tumor tissue, as reflected by a p-value of 1.62436730732907E-12.

Table S1. List of miRNAs Predicted to be Sponged by Selected circRNAs Based on circInteractome Data

**hsa_circ_0002599**	**hsa_circ_0088251**	**hsa_circ_0044556**	**hsa_circ_0001875**	**hsa_circ_0001414**
hsa-miR-1272	hsa-miR-100	**hsa-miR-145-5p**	hsa-miR-1180	hsa-miR-100
hsa-miR-136	hsa-miR-1180	hsa-miR-197	hsa-miR-1184	hsa-miR-1179
hsa-miR-145	hsa-miR-1208	hsa-miR-338-3p	hsa-miR-1200	hsa-miR-1225-5p
hsa-miR-194	hsa-miR-1224-3p	hsa-miR-589	hsa-miR-1205	hsa-miR-1251
**hsa-miR-326-3p**	hsa-miR-1225-5p	hsa-miR-615-5p	hsa-miR-1257	hsa-miR-1261
hsa-miR-330-5p	hsa-miR-1252	hsa-miR-634	hsa-miR-1287	hsa-miR-1305
hsa-miR-510	hsa-miR-1282	hsa-miR-665	hsa-miR-1296	hsa-miR-145
hsa-miR-619	hsa-miR-1304	hsa-miR-766	hsa-miR-1299	hsa-miR-183
hsa-miR-634	hsa-miR-1307		**hsa-miR-145-5p**	hsa-miR-203
hsa-miR-658	hsa-miR-140-3p		hsa-miR-151-3p	hsa-miR-338-5p
hsa-miR-767-3p	hsa-miR-149		hsa-miR-182	hsa-miR-421
hsa-miR-892b	hsa-miR-1827		hsa-miR-1825	hsa-miR-490-5p
hsa-miR-942	hsa-miR-183		hsa-miR-183	hsa-miR-494
	**hsa-miR-326-3p**		hsa-miR-203	hsa-miR-558
	hsa-miR-330-5p		hsa-miR-296-5p	hsa-miR-561
	hsa-miR-377		hsa-miR-31	hsa-miR-569
	hsa-miR-513a-5p		hsa-miR-384	hsa-miR-576-5p
	hsa-miR-516b		hsa-miR-409-3p	hsa-miR-604
	hsa-miR-556-5p		hsa-miR-488	hsa-miR-605
	hsa-miR-625		hsa-miR-516b	hsa-miR-616
	hsa-miR-636		hsa-miR-545	hsa-miR-647
	hsa-miR-640		hsa-miR-548p	hsa-miR-659
	hsa-miR-653		hsa-miR-570	hsa-miR-767-5p
	hsa-miR-657		hsa-miR-671-5p	hsa-miR-885-3p
	hsa-miR-671-5p		hsa-miR-889	**hsa-miR-99a**
	hsa-miR-873		hsa-miR-891b	hsa-miR-99b
	hsa-miR-874		hsa-miR-921	
	hsa-miR-892b			
	hsa-miR-924			
	hsa-miR-99a			
	hsa-miR-99b			

The table presents the list of miRNAs predicted to be sponged by each selected circRNAs, identified using circInteractome tool. Each column corresponds to a specific circRNA and lists its proposed miRNAs. The miRNAs with font color red are those specifically proposed and addressed in the review paper as potential sponging targets of the proposed circRNAs. Additionally, shared miRNAs, predicted to be sponged by multiple circRNAs, are highlighted with same color across columns to visually indicate the common ones.
